# Impact of the COVID-19 pandemic on hospital admissions for cardiovascular diseases in a large Brazilian urban center

**DOI:** 10.1590/0037-8682-0264-2021

**Published:** 2022-01-28

**Authors:** Edmar Geraldo Ribeiro, Pedro Cisalpino Pinheiro, Bruno Ramos Nascimento, João Pedro Pereira Cacique, Renato Azeredo Teixeira, Jamil de Souza Nascimento, Tulio Batista Franco, Luisa Campos Caldeira Brant, Deborah Carvalho Malta

**Affiliations:** 1 Universidade Federal de Minas Gerais, Escola de Enfermagem, Departamento Materno Infantil, Belo Horizonte, MG, Brasil.; 2 Universidade Federal de Minas Gerais, Faculdade de Medicina, Belo Horizonte, MG, Brasil.; 3 Ministério da Saúde, Superintendência Estadual do Ministério da Saúde em Minas Gerais. Belo Horizonte, MG, Brasil.; 4 Universidade Federal Fluminense, Departamento de Planejamento em Saúde, Rio de Janeiro, RJ, Brasil.

**Keywords:** Cardiovascular diseases, Hospitalization, COVID-19, Time series studies, Health systems, Epidemiology

## Abstract

**INTRODUCTION:**

The COVID-19 pandemic has had a great impact on the behavior of individuals and the organization of health systems. This study analyzed the COVID-19 pandemic’s effect on public hospitalizations for cardiovascular diseases (CVD) in a large city in Brazil, Belo Horizonte, MG, with approximately 2.5 million inhabitants.

**METHODS:**

In a time-series analysis, this study used administrative data from the national “Hospital Information System” from 2010 to February 2020 to estimate the expected number of hospitalizations for CVD by month during the COVID-19 pandemic in Belo Horizonte in 2020 using the Auto-Regressive Integrated Moving Average model. For CVD, this study compared the expected number of hospital admissions, intensive care use, deaths during hospitalization, and mean length of stay with the observed number during the period.

**RESULTS:**

There were 6,517 hospitalizations for CVD from March to December 2020, a decrease of 16.3% (95% CI: 4.7-25.3) compared to the projected. The number of intensive care hospitalizations for CVD fell 24.1% (95% CI 13-32.7). The number of deaths also decreased (17.4% [80% CI: 0 - 0.30]), along with the reduction in hospitalizations, as did the length of stay for CVD hospitalizations. These reductions, however, were not significant.

**CONCLUSIONS:**

Hospitalizations for CVD were 16.3% lower than expected in a large Brazilian city, possibly due to the fear of getting infected or going to hospitals. Public campaigns informing how to proceed in case of CVD show that prompt urgent attention is essential to mitigate the indirect effects of the pandemic on CVD.

## INTRODUCTION

Cardiovascular diseases (CVD) are the leading causes of death worldwide and are responsible for the loss of 17.9 million lives each year, of which one third occur prematurely in people under the age of 70^1^. According to the Brazilian Unified Health System (Sistema Único de Saúde, SUS, in Portuguese) databases, CVDs are also responsible for considerable morbidity. In addition to being the cause of most disability pensions, they are also associated with high rates of hospitalization and a progressive increase in hospital costs, most likely due to the increased complexity of treatments^2^ ^,^ ^3^. According to data from the Hospital Information System (Sistema de Informação Hospitalar, SIH-DATASUS, in Portuguese), CVDs that resulted in the highest number of hospitalizations in Brazil in the past 10 years were, in this order: heart failure (HF), stroke, and Acute Coronary Syndromes (ACS)^4^.

In 2020, with the coronavirus pandemic (COVID-19), social distancing policies were adopted worldwide, and health systems were reorganized to deal with the increasing number of patients infected with Coronavirus 2 Severe Acute Respiratory Syndrome (SARS-CoV-2)^5^, prioritizing such services. During the same period, studies in high- and low-income countries showed changes in the pattern of hospital admissions with an unexpected decline in admissions for CVD^6^ ^,^ ^7^ ^,^ ^8^. Studies in the city of São Paulo and in Brazil for the first five months of the pandemic also showed a decline in hospital admissions^6^ ^,^ ^7^. Given the known deleterious effects of COVID-19 on the cardiovascular system, it is important to analyze whether these unexpected declines occurred in other locations, as we would expect an opposite trend, since SARS-CoV-2 leads to an inflammatory response and predisposes the patient to thrombotic events, which could ultimately result in a greater demand for hospitalization due to CVD^9^. 

The present study aims to analyze the impact of the COVID-19 pandemic on hospital admissions for CVD in a large city from a middle-income country (Belo Horizonte, Minas Gerais, Southeast Brazil) in 2020.

## METHODS

This is a time-series study, using data from SIH-DATASUS^4^ for hospitalizations in Belo Horizonte - a large urban center in Brazil, with approximately 2.5 million inhabitants - due to the following causes: HF, considering hospitalizations with a main diagnosis of I50, according to the 10^th^ revision of the International Classification of Diseases (ICD-10) codes; stroke, based on hospitalizations with codes I60 - I64; ACS, codes I20 - I24; and CVD, which includes the 3 previous groups. The following stratified variables were analyzed for each cause: number of hospitalizations, number of hospitalizations with Intensive Care Unit (ICU) admissions, number of hospitalizations resulting in death, and average length of hospital stay. In addition, analyses were stratified by age, considering the 60 years of age cutoff (adults and older adults), and by sex. Data were analyzed until December 2020. 

The Auto-Regressive Integrated Moving Average (ARIMA) for a time-series model was used to estimate the expected number of the variables of interest within the period, from March to December 2020, which serves as a hypothetical scenario for comparison with data observed during the same period. ARIMA is a combination of an autoregressive model with differentiation and a moving average model^10^. For the projection of the indicators in the study period, a monthly time series was built for the variables of interest from January 2010 to December 2020. However, for a better understanding and visualization, the results from January 2015 to December 2020 were presented. February 2020 was defined as the last month in which the trends for study variables would not have been influenced by the COVID-19 pandemic, considering that the Belo Horizonte City Hall (Prefeitura de Belo Horizonte, PBH, in Portuguese) issued social isolation policies, closing schools and other non-essential services, in March 2020^11^. The months from March to December 2020 were not considered in the model estimates, because changes observed during this period would affect the estimated model and, consequently, the projections. All statistical analyses were performed, using R (R Core Team, 2020)^12^, together with the fpp2 package^13^. In the supplementary section, we present a table with the final estimated model for each variable and subgroup (**Table 01**). Autocorrelation Function (ACF) and Partial Autocorrelation Function (PACF) figures for each model are also available in the supplementary section. More details on how the time-series was modeled can be found on the package website (https://otexts.com/fpp2/arima-r.html). 

Once models and projections for each of the analyzed series were estimated, expected and observed indicators were compared. For the total number of hospitalizations, ICU admissions, and deaths, this study compared the observed sum between March and December, with the same sum based on the projection of the time series for 2020. For the average number of hospital days, the average of the period observed was compared with the average of the projected period. Calculations were performed considering a percent variation, that is, the ratio between observed and projected, subtracted by 1. To incorporate the uncertainty of the projection in the analysis, the upper and lower limits of the projection’s prediction intervals (80% and 95%) were also used for comparisons between scenarios.

The SIH-DATASUS information was obtained in an aggregated form, without patient identification, so there was no need for submission to an Institutional Review Board (IRB). The study was conducted in accordance with Resolution No. 510 of April 7, 2016 of the National Health Council (Conselho Nacional de Saúde, CNS, in Portuguese). 

## RESULTS

**FIGURE 1:**
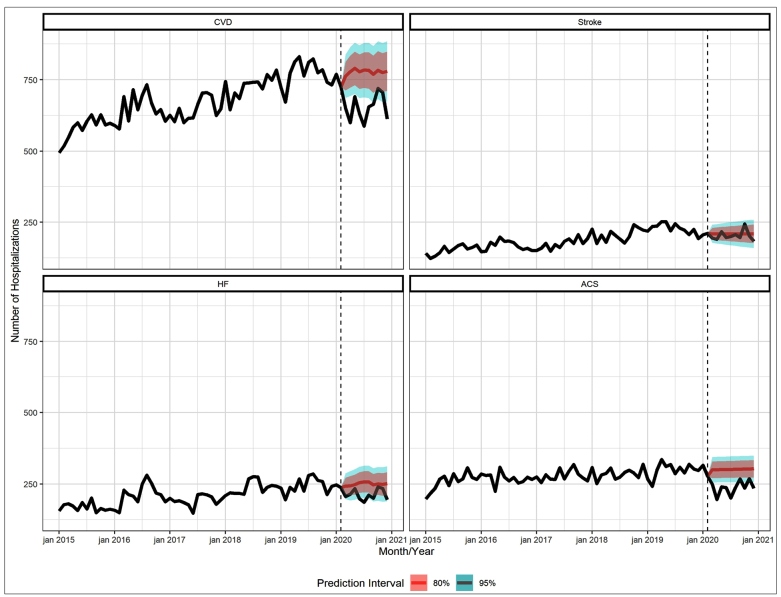
Time-series, from January 2015 to December 2020, with the number of observed and expected hospitalizations, and the 80% and 95% prediction intervals.

Figure 1 shows the time-series, from January 2015 to December 2020, with the number of observed and expected hospitalizations, as well as the 80% and 95% prediction intervals, for: CVD, stroke, HF, and ACS, for both sexes and all ages. There was a significant reduction in the number of hospitalizations for CVD. CVD hospitalizations were reduced in 16.3% in the pandemic period of 2020, compared to what was expected for the period. The series of hospitalizations due to ACS (Figure 1), which had less variability in the period between January 2015 and February 2020, depicts the difference between the observed and expected scenarios. For example, the average projection scenario estimates 3,013 hospitalizations for ACS in the 2020 pandemic period, while only 2,369 admissions due to this cause actually occurred.

**FIGURE 2:**
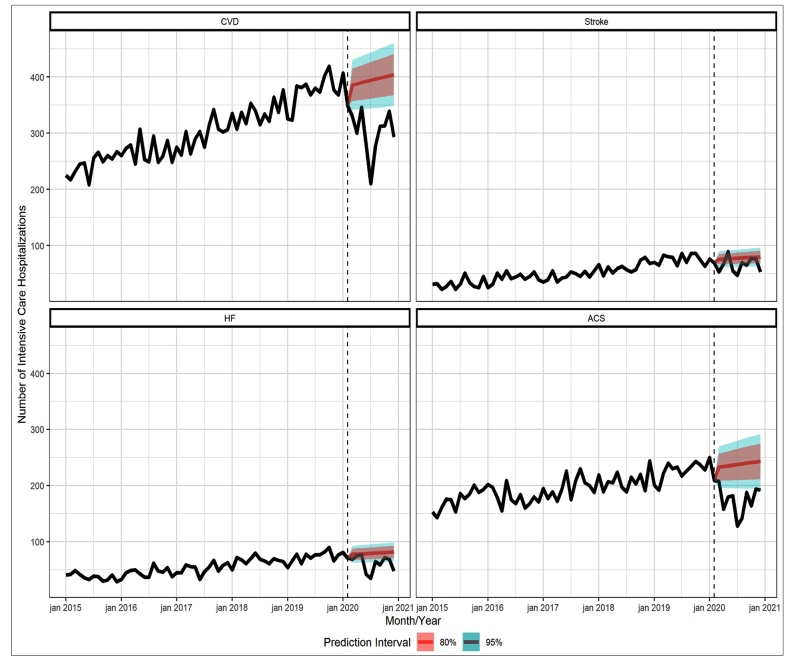
Time-series, from January 2015 to December 2020, with the number of observed and expected hospitalizations with ICU admissions, and the 80% and 95% prediction intervals.

Figure 2 shows the time-series with the number of observed and expected ICU admissions, with prediction intervals, for CVD, stroke, HF, and ACS. Similar to the total number of hospitalizations, those with ICU stay showed a significant and continuous drop after February 2020. Interestingly, there was an increase in the number of ICU admissions between 2015 and the beginning of 2020, for all outcomes, markedly for total CVD. In the months following February, the upward trend reversed. Considering total CVDs, hospitalizations with ICU stay fell 24.1% (95% CI: 13-32.7) during the 2020 pandemic period.

**FIGURE 3:**
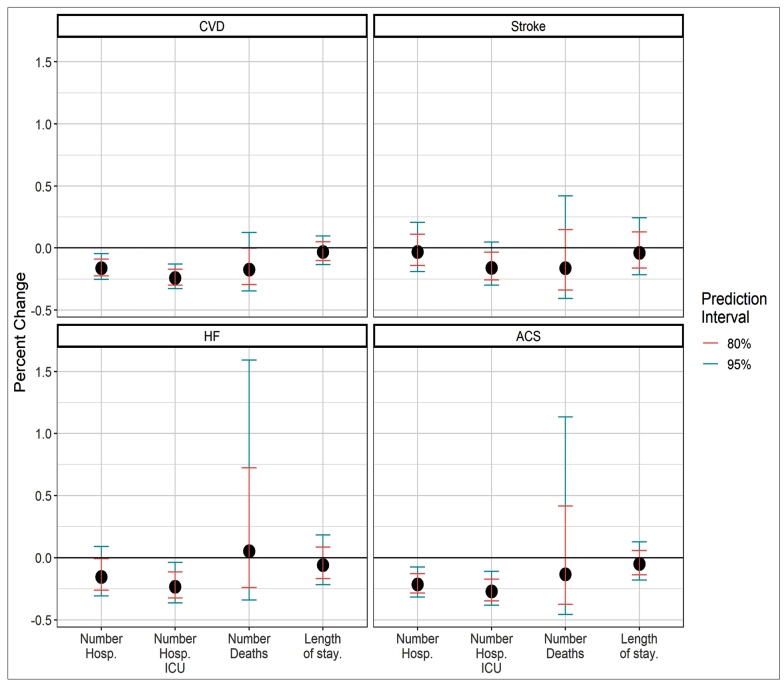
Comparison between the observed and expected scenarios for all outcomes and analyzed variables: number of hospitalizations, number of hospitalizations with ICU admission, in-hospital deaths, and length of hospital stay.

Figure 3 presents the comparison between the observed and expected scenarios for all outcomes and analyzed variables. Considering CVD, the variation indicates that the observed variables showed a proportional decrease, except for the average length of hospital stay. The most expressive reductions were observed for the number of hospitalizations and the total number of those requiring ICU admissions. For the number of in-hospital deaths, the reduction was only significant for the 80% prediction interval (17.4% [80% CI: 0% - 0.30%]). For ACS, the trends were similar, while for HF and stroke, the variation was not significant for the analyzed variables, except for the number of ICU hospitalizations, since HF was significantly lower than expected. 

Table 1 shows the proportional variation between observed and expected scenarios, for CVD, stroke, HF, and ACS, stratified by sex and age (cutoff of 60 years of age). The values indicate the variation based on the average projection scenario and variation is presented in the parentheses, considering the upper and lower limits (95%) of the prediction interval, respectively. Considering the number of hospitalizations for CVD, a significant decline was observed for all subgroups, except for men under 60 years of age, as well as a decrease in hospitalizations for ACS in older adults for both sexes. Regarding ICU admissions, it is worth noting that the reduction was significant for CVD, HF, and ACS only for older adults.

**TABLE 1:** Percentage variation between observed and projected (ARIMA) Hospitalizations, CVD, Stroke, HF, ACS, March - December 2020.

	Both Sexes			Female			Male		
	Obs	Exp	% Variation	Obs.	Fore.	% Variation	Obs.	Fore.	% Variation
**Number of Hospitalizations**										
CVD	6517	7782.8	-16.3	2980	3606.6	-17.4	3537	4262.5	-17
All ages			(-4.7;-25.3)*			(-3.3;-27.9)*			(-4.8;-26.5)*
CVD	2118	2482.1	-14.7	825	1081.2	-23.7	1293	1425.8	-9.3
0-59y			(1.1;-26.2)			(-5.3;-36.1)*			(13.2;-24.4)
CVD	4399	5363.2	-18	2155	2592.2	-16.9	2244	2783.1	-19.4
60+y			(-5.8;-27.4)*			(-1.1;-28.3)*			(-5.5;-29.7)*
Stroke	2029	2095.7	-3.2	985	1139.1	-13.5	1044	1038.6	0.5
All ages			(20.4;-19.1)			(6.2;-27.1)			(34;-19.6)
Stroke 0-59	625	757.1	-17.5	281	386.1	-27.2	344	348.6	-1.3
			(8.1;-33.2)			(4.1;-44.1)			(55.3;-27.7)
Stroke - 60+	1404	1390.2	1	704	735.0	-4.2	700	763.7	-8.3
			(30;-17.4)			(25.6;-22.6)			(20.8;-26.2)
HF	2119	2501.5	-15.3	1080	1284.6	-15.9	1039	1170.9	-11.3
All ages			(9;-30.7)			(17.2;-34.4)			(19.4;-29.4)
HF - 0-59	515	657.1	-21.6	198	315.2	-37.2	317	347.2	-8.7
			(9;-38.8)			(-6.2;-52.8)*			(44.6;-33.3)
HF - 60+	1604	1880.1	-14.7	882	961.5	-8.3	722	855.5	-15.6
			(11.3;-30.8)			(37.1;-31.1)			(14.5;-33.2)
ACS	2369	3013.9	-21.4	915	1168.0	-21.7	1454	1832.8	-20.7
All ages			(-7.5;-31.7)*			(3.2;-36.9)			(-2.7;-33)*
ACS - 0-59	978	1116.3	-12.4	346	378.8	-8.7	632	753.1	-16.1
			(14.7;-29.1)			(42.1;-32.7)			(14.7;-33.8)
ACS - 60+	1391	1927.6	-27.8	569	796.8	-28.6	822	1133.2	-27.5
			(-12.4;-8.7)*			(-5.2;-42.7)*			(-8.9;-39.7)*
**Number of ICU Hospitalizations**										
CVD	2999	3951.3	-24.1	1288	1677.5	-23.2	1711	2294.5	-25.4
All ages			(-13;- 32.7)*			(-8;-34.1)*			(-12;-35.3)*
CVD - 0-59	1115	1381.2	-19.3	424	535.1	-20.8	691	841.5	-17.9
			(2;-33.2)			(10.3;-38.2)			(7.5;-33.6)
CVD - 60+	1884	2654.0	-29	864	1141.5	-24.3	1020	1445.9	-29.5
			(-20.2;-36.1)*			(-7.7;-35.8)*			(-15.7;-39.4)*
Stroke	652	776.8	-16.1	322	390.8	-17.6	330	416.6	-20.8
All ages			(4.8;-30)			(12.9;-35.2)			(5;-36.4)
Stroke - 0-59	242	312.0	-22.4	114	156.6	-27.2	128	155.1	-17.5
			(14.8;-41.4)			(42.7;-51.1)			(40.2;-41.5)
Stroke - 60+	410	455.7	-10	208	234.4	-11.3	202	233.3	-13.4
			(20.9;-28.3)			(30.2;-32.7)			(29.1;-34.9)
HF	611	796.2	-23.3	303	407.5	-25.6	308	396.3	-22.3
All ages			(-3.7;-36.2)*			(0.1;-40.8)			(5.5;-38.5)
HF - 0-59	150	210.7	-28.8	56	91.0	-38.5	94	115.0	-18.3
			(11;-47.6)			(22.4;-58.9)			(53;-44.3)
HF - 60+	461	592.8	-22.2	247	304.7	-18.9	214	279.3	-23.4
			(-1.7;-35.7)*			(16.5;-37.8)			(5.5;-39.9)
ACS	1736	2379.4	-27	663	889.8	-25.5	1073	1502.6	-28.6
All ages			(-10.9;-38.2)*			(-0.5;-40.4)*			(-10.5;- 40.6)*
ACS - 0-59	723	834.0	-13.3	254	280.1	-9.3	469	557.4	-15.9
			(22.1;-32.8)			(53.9;-35.7)			(24.2;-36.4)
ACS - 60+	1013	1531.9	-33.9	409	601.8	-32	604	923.7	-34.6
			(-18.7;-44.3)*			(-6.9;-46.5)*			(-15.8;-46.5)*

*Negative variation even considering the lower bond of the prediction interval.

In parentheses percent variation estimated using, respectively, upper and lower of the 95% prediction Interval.

When the number of hospitalizations resulting in death and the average number of hospital days were analyzed, no significant difference was found for any of the subgroups (data not shown).

## DISCUSSION

During the COVID-19 pandemic in 2020, a decline in the number of hospital admissions and in the number of individuals admitted to ICU beds for total CVD and for ACS was identified in Belo Horizonte, when compared to the historical series since 2015. For total CVD, this decline occurred for both sexes in older adults, and for women when considering younger adults (<60y) and number of hospitalizations. For hospitalizations due to ACS, the reduction was significant only for individuals over 60 years of age, suggesting that the impact on ICU admissions for CVD was differential according to the age group, revealing more unfavorable trends for older adults. Regarding hospitalizations resulting in death, although we observed a reduction in the number of hospital admissions, there was a non-significant absolute reduction for in-hospital deaths, in parallel with the reduction in hospitalizations, possibly due to a greater severity of hospitalized individuals^6^. As for the average length of hospital stay, no significant difference was found when compared to the historical series. We hypothesized that this resulted from two opposing trends: reducing hospital days as much as possible due to the risk of exposure to COVID-19 and increasing hospital stay due to greater severity of hospitalized patients. Taken together, our data reveal a major impact of the COVID-19 pandemic on hospital admissions for CVD, showing that the effects of the pandemic are beyond those directly caused by viral infection.

The reduction in hospitalizations for CVD has also been described in other countries, such as Italy, Spain, Austria, the United States, and China^5^ ^,^ ^8^ ^,^ ^14^ ^,^ ^15^ ^,^ ^16^. In Northern Italy, the number of hospitalizations for ACS decreased 28% between February 20 and March 31, 2020^5^. In California (US), the weekly hospitalization rate for acute myocardial infarction also decreased from March 4 to April 14 by up to 48%^17^. In China, stroke admissions decreased by 40% in February 2020 as compared to 2019. The rate of fibrinolysis and thrombectomy procedures also decreased, but to a lesser extent (25%)^18^. During this period, authors reported a decline in hospital capacity dedicated to stroke treatment of 50% in most institutions^19^. In addition to the reorganization of health systems - with the deactivation of services to meet urgent needs or to increase intensive capacity for patients infected with SARS-COV2, the delimitation of specific hospitals for COVID-19, and the implementation of alternative therapeutic pathways^20^ - there are also other explanations for the phenomenon of reduction of hospitalizations for CVD observed in several countries. Due to social distancing regulations and to the fear of contracting COVID-19, individuals sought out health care less frequently, especially those with CVD or risk factors, as they were part of the high-risk population for mortality^21^ ^,^ ^22^. In addition, isolation may make it difficult for others to detect cardiovascular symptoms, and therefore not transport patients to a hospital in a timely manner^23^.

It is important to highlight that, in parallel with the reduction in hospitalizations due to CVD during the pandemic, an excess mortality was also observed due to natural causes in several locations^24^ ^,^ ^25^. Excess mortality measures the number of deaths that exceeds those expected for a given period based on the historical series, thus reflecting direct and indirect effects of the pandemic on the mortality of a given population^26^. Studies suggest that SARS-COV2 infection alone may not explain all beyond-expected deaths that occurred during the pandemic^27^. Analyzing causes of deaths that occurred during the pandemic in the US from March 1 to April 25, only 65% ​​were directly attributed to COVID-19, while the other main causes were: diabetes, Alzheimer's disease, and CVD^28^. In addition, in Italy, there was a 58% increase in out-of-hospital sudden deaths between February 21 and March 31, 2020, compared to 2019, suggesting that more deaths from CVD may have occurred before individuals were admitted to hospitals^29^.

A recent study evaluating the excess of cardiovascular mortality during the COVID-19 pandemic in six Brazilian capitals found that CVD mortality increased in most cities, markedly in Northern capitals^30^. However, in more developed cities, there was a reduction in ACS and stroke deaths, and the excess of CVD deaths occurred only due to the increase in unspecified cardiovascular events^30^. It is important to emphasize that this increase in unspecified cardiovascular deaths correlated with the increment in home deaths, suggesting that CVD deaths may have occurred without health care, and therefore lack a definitive diagnosis^30^. In addition, Brant et al. showed that excess CVD mortality was higher in less developed cities, which may be related to the transport infrastructure, difficulties to access healthcare services, or a collapse in the health system^30^.

In the context of the aforementioned data, the present study suggests that the reduction in hospitalizations for CVD may be an indirect effect of the pandemic. The hypothesis is that individuals sought out less hospital care for acute or chronic decompensated CVD, but this did not result in a reduction in CVD in-hospital mortality. In fact, there may have been an increase in the number of CVD overall deaths, but without a definite diagnosis due to a lack of medical care. It is worth noting that the indirect effects of the pandemic on CVDs may last for years, due to the postponement or interruption of primary and secondary preventive measures for CVD during the pandemic, and the increase in cardiovascular risk factors during this period (such as physical inactivity and the consumption of alcohol, tobacco, and processed foods), which have long-term effects on CVD^22^ ^,^ ^31^ ^,^ ^32^. A virtual study conducted by the Pan American Health Organization (PAHO), carried out in 158 countries, showed that preventive services and treatment of chronic non-communicable diseases (NCDs) were seriously affected, posing a threat to the population with CVD^33^. Furthermore, another study revealed that the volume of diagnostic procedures for CVD decreased by 42% from March 2019 to March 2020, and 64% from March 2019 to April 2020 in 108 countries, and these procedures are key to effective treatment guidance. This issue worsens the impacts on the care of patients with cardiovascular risks^34^.

## LIMITATIONS

The present study has limitations. As a retrospective observational study, with data derived from a national database (HIS-DATASUS)^4^, it is possible that there was underreporting, notification delays, or erroneous data entry. However, analyzing the historical series, data about hospitalizations in a certain month are, mostly (average 98.3%) processed within four months. In 2018 and 2019, on average, only 1.7% of admissions were processed after the fourth month (not counting the month of interest), reinforcing our findings. In 2020, the processing period followed the same path. We used data processed until April 2021, which minimizes the possibility of underreporting in the analysis period. In addition, this is a single-city study of public hospitalizations, and it is well-known that the impact of the pandemic is heterogeneous between locations, which hinders the generalization of our data to other Brazilian municipalities. However, data from other regions of the world have already depicted similar trends, supporting our findings.

The impact of the COVID-19 pandemic resulted in changes in the scenario of hospitalizations due to CVD, with a decrease in the use of healthcare services. Public awareness efforts about acute cardiovascular symptoms requiring immediate hospital care, even during social isolation, as well as informative campaigns about the reorganization of healthcare services, must be carried out and encouraged. In addition, the resumption of health promotion and preventive measures for CVDs must adapt to the new reality and be implemented as soon as possible so as to manage the increased cardiovascular risk of individuals during the pandemic, in an attempt to minimize its deleterious effects on cardiovascular health.

## CONCLUSION

During the first months of the COVID-19 pandemic, a reduction in the number of CVD admissions was observed in Belo Horizonte, as compared to the historical series since 2015. This reduction in the total number of hospitalizations and those requiring ICU stay for CVD may have presumably occurred due to social isolation, reduced access to healthcare services, fear of contamination, among others. Failure to seek out health care raises concerns about the clinical evolution and prognosis of patients with CVD during pandemic times. Public awareness campaigns about the importance of seeking medical care in case of acute cardiovascular symptoms, explanation about new healthcare flows, in addition to the resumption of measures for health promotion and cardiovascular risk factor controls, should be prioritized.
